# Incidence of pneumococcal disease from 2003 to 2019 in children ≤17 years in England

**DOI:** 10.1186/s41479-022-00103-3

**Published:** 2023-01-23

**Authors:** Salini Mohanty, Bélène Podmore, Ana Cuñado Moral, Ian Matthews, Eric Sarpong, Alessandra Lacetera, Nawab Qizilbash

**Affiliations:** 1 Merck & Co., Inc, Center for Observational and Real-World Evidence (CORE), Rahway, NJ USA; 2 OXON Epidemiology Ltd, Epidemiology & Statistics, Madrid, Spain; 3 MSD (UK) Ltd, Value, Access and Devolved nations (VAD), London, UK; 4 Merck & Co., Inc., Real-world Data Analytics and Innovation (RDAI), Rahway, NJ USA

**Keywords:** Invasive pneumococcal disease, Pneumococcal disease, Pneumococcal pneumonia, Pneumococcal conjugate vaccine, United Kingdom

## Abstract

**Background:**

Pneumococcal disease is a leading cause of communicable disease morbidity and mortality globally. We aimed to estimate invasive pneumococcal disease (IPD), pneumococcal pneumonia (PP) and all-cause pneumonia (ACP) incidence rates (IRs) in children aged 0–17 years in England from 2003 to 2019.

**Methods:**

A retrospective study in children ≤17 years old from 2003 to 2019 using the Clinical Practice Research Datalink (CPRD) Gold and Hospital Episodes Statistics Admitted Patient Care (HES APC) databases. IPD episodes were identified in hospital records (HES APC). PP (caused by *Streptococcus pneumoniae* only) and ACP episodes (caused by any pathogen) were identified in primary care (CPRD) and in hospital records (HES APC). Annual IRs by age-group were calculated as the number of episodes/person-years (PY) at risk, with 95% confidence intervals (95% CI). Interrupted time series analyses were conducted to assess changes in IRs across the post-PCV7 (2007–2009), early post-PCV13 (2011–2014) and late post-PCV13 (2015–2019) periods compared to the pre-PCV7 period (2003–2005) using generalized linear models.

**Results:**

170 IPD episodes, 769 PP episodes and 12,142 ACP episodes were identified in 1,500,686 children in 2003–2019. The overall IPD, PP and ACP IRs (per 100,000 PY) were 2.29 (95% CI 1.96–2.66), 10.34 (95% CI 9.62–11.10) and 163.37 (95% CI 160.47–166.30), respectively. The highest IPD, PP and ACP IRs were observed in children aged < 2 years compared to older children (2–4 and 5–17 years). IPD IRs decreased between the pre-PCV7 period and the late post-PCV13 period from 3.28 (95% CI 2.42–4.33) to 1.41 (95% CI 0.80–2.29), IRR 0.28 (95% CI 0.09–0.90), *p*-value 0.033. PP IRs declined between the pre-PCV7 period and the late post-PCV13 period from 14.65 (95% CI 12.77–16.72) to 3.87 (95% CI 2.81–5.20), IRR 0.19 (95% CI 0.09–0.38), *p*-value < 0.001. ACP IRs declined between the pre-PCV7 period and the late post-PCV13 period from 167.28 (95% CI 160.78–173.96) to 124.96 (95% CI 118.54–131.63), IRR 0.77 (95% CI 0.66–0.88), p-value < 0.001.

**Conclusions:**

The clinical burden of IPD, PP and ACP declined in children in England aged 0–17 years between 2003 and 2019, especially in the late post-PCV13 period. This study highlights the importance of PCV vaccination in reducing the burden of PD and ACP in children in England.

**Supplementary Information:**

The online version contains supplementary material available at 10.1186/s41479-022-00103-3.

## Background

Pneumococcal disease is caused by the bacterium *Streptococcus pneumoniae* (*S. pneumoniae*) [1]. When *S. pneumoniae* is isolated from the blood or another normally sterile site, it is referred to as invasive pneumococcal disease (IPD) and includes infections such as bacteremia, bacteremic pneumonia, meningitis, and sepsis. Non-invasive pneumococcal disease includes infections such as non-bacteremic pneumococcal pneumonia, otitis media, and sinusitis [1, 2]. *S. pneumoniae* is the most common cause of bacterial pneumonia in children [3]. The Global Burden of Diseases (GBD) Study 2016 provided an analysis of the burden of lower respiratory infections (LRI) in 195 countries [4]. The incidence of bacterial LRI in children aged < 5 years was highest for *S. pneumoniae* [70.7 (95% uncertainty interval, UI 33.0–116.6) per 1000 people] compared to *Haemophilus influenzae type b*, 9.6 (95% UI 2.2–21.7) per 1000 people [4].

Pneumococcal disease is one of the leading causes of communicable disease morbidity and mortality in Europe and globally, with the highest burden of disease found in young children and the elderly [1]. According to surveillance data from the European Surveillance System (TESSy) in 2018, the reported IPD incidence was 14.4 confirmed cases per 100,000 population in infants aged < 1 year old [5].

In 2006, the UK introduced the 7-valent pneumococcal conjugate vaccine (PCV7) into the routine childhood immunization program at two and four months, providing protection against serotypes 4, 6B, 9 V, 14, 18C, 19F, and 23F, followed by a booster after the first birthday [6, 7]. From April 2010, the 13-valent PCV (PCV13) replaced PCV7, and was administered at 8 and 16 weeks, with a booster at 12–13 months of age [8, 9]. In April 2019, the Joint Committee on Vaccination and Immunisation (JCVI), advised following a 1 + 1 schedule for all infants born on, or after 1 January 2020. This schedule includes a single dose of PCV13 administered at 12 weeks of age, followed by a PCV13 booster at 12 months (on or after the first birthday), thereby switching from a 2 + 1 to 1 + 1 schedule [7, 10, 11].

Previous studies in England and Wales assessing the effect of PCV7 and PCV13 introduction have reported a reduction in IPD incidence rates (IRs). A study by Waight PA., et al. included IPD cases among children and adults in surveillance data from July 2013 and June 2014 and reported a 56% overall reduction after the replacement of PCV7 with PCV13 in April 2010 when compared with the pre-PCV7 baseline [12]. Ladhani SN., et al. using surveillance data for 2016/17, reported that IPD incidence across all ages was 37% lower than pre-PCV7 incidence and 7% lower than pre-PCV13 incidence [6].

Previous studies reporting on the effects of PCV7 and PCV13 on the IRs of pneumococcal pneumonia (PP) and all-cause pneumonia (ACP) have been less conclusive, and recent estimates are lacking. Thorrington D., et al., using hospital admissions data, reported a decline in hospital admissions for IPD and PP for children aged < 15 years (< 2, 2–4 and 5–14-year-old age groups) [13]. The largest reductions of hospital admissions for IPD and PP from 2004 to 2015 were in children aged < 2 years. ACP IRs reported in previous studies in the UK have varied significantly due to the variation in methodology and populations studied. Lau WCY., et al. using the IMS Disease Analyser database reported a decline in the overall annual ACP IRs of 37% from 2002 to 2012 in children aged < 10 years [14]. Sun X., et al. reported a decrease in ACP IRs over a similar study period in children aged < 15 years using the Clinical Practice Research Datalink (CPRD) database [15]. In contrast, Saxena S., et al. conducted a study using hospital admissions data in England, from 2001 to 2014 for children < 16 years, and reported no added benefit of PCV13 over PCV7 on ACP admissions following PCV13 introduction [16].

According to Waight PA., et al., the herd protection induced by PCV7 and PCV13 persists in England and Wales [12]. However, there was evidence of increasing IPD due to non-PCV13 serotypes, particularly in children under 5 years of age in 2014 [12]. Higher-valent PCVs are now in late-stage clinical development for paediatric use, including a 15-valent PCV (PCV15) recently approved for use in adults and children in Europe, the US and Canada [17–20], and a 20-valent PCV (PCV20) approved for use in adults in Europe [21] and the US [22]. Quantifying the incidence and burden of pneumonia and IPD following the most recent years after PCV13 introduction is essential prior to the introduction of higher-valent PCVs. The aim of this study is to provide recent estimates of PP, ACP and IPD incidence rates in children aged 0–17 years in England from 2003 to 2019.

## Methods

### Study design

A retrospective observational cohort study was conducted using linked data from the CPRD-Gold and the Hospital Episodes Statistics Admitted Patient Care (HES APC) databases [23, 24]. The study included children aged ≤17 years in England from 1 January 2003 to 31 December 2019. To be eligible for inclusion, children aged 1–17 years needed to have: (1) at least 12 months of medical up-to-standard practice data [UTS; measure of data quality as defined in CPRD [24]], (2) last practice data collection date and (3) study inclusion date to ensure their medical history could be assessed with continuous follow-up in the last 6 months. Continuous follow-up was defined from the current registration date and only for patients with no follow-up interruptions or single interruptions ≤7 days. Children aged < 1 year did not need to meet these criteria. The start of the follow-up period was defined from the earliest of these events: (i) start of study period (1 January 2003), (ii) date of birth, or (iii) start of data collection. The end of the study period was defined by the earliest of the following events (i) end of study period (31 December 2019), (ii) end of year in which patient turns 17 years, (iii) death, (iv) transfer out of practice, or (v) end of data collection.

### Data source

Children were identified using the CPRD-Gold database linked to the HES APC database. The CPRD-Gold is a database comprising anonymized medical records from primary care practices in England as part of routine clinical care [24]. With 985 practices and 3.11 million current acceptable patients (i.e., registered at currently contributing practices, excluding transferred out and deceased patients), 4.64% of the UK population is covered [25] and patients are representative of the general population in the UK in terms of age, sex and ethnicity [24]. As general practitioners are responsible for primary care and referrals to specialists in the UK, the CPRD primary care database is therefore a reliable source of health data for research, and includes data on demographics, symptoms, diagnoses, tests, health-related behaviors, therapies and referrals to secondary care [24]. The HES APC database includes all inpatient admissions to National Health Service (NHS) hospitals in England since 1997. HES APC contains information on primary and secondary diagnoses, procedural events, and dates of admission and discharge from hospital [23].

### Outcomes & Covariates

An IPD episode included episodes caused by *S. pneumoniae* including pneumococcal bacteremia/septicemia, pneumococcal meningitis, pneumococcal bacteremic pneumonia and other IPD manifestations (e.g., pericarditis) [1, 2, 13, 26]. IPD inpatient episodes were identified using the International Statistical Classification of Diseases and Related Health Problems 10^th^ revision (ICD-10) diagnosis codes in the HES APC database (see Supplementary Table 1).

Two pneumonia definitions were used: (1) PP episodes caused by *S. pneumoniae,* and (2) ACP episodes caused by *S. pneumoniae*, presumed to be caused by *S. pneumoniae* or by all other unknown and known pathogens. Pneumonia episodes were identified in primary care and inpatient settings through linked CPRD-Gold and HES APC data using Read diagnosis codes and ICD-10 diagnosis codes, respectively (see Supplementary Table 1).

A gap of 90 days between an episode with IPD/pneumonia diagnosis codes defined the start of a new episode [13, 26]. For GP registered episodes in which there was only a single visit with a Read diagnosis code for pneumonia, a period of 14 days after that visit was considered to account for antibiotic treatment.

The population characteristics that were described included the following demographic factors: age group, sex, geographic region, urbanicity, social deprivation, ethnicity and risk factors. Urbanicity was defined using the Rural-Urban classification, which distinguishes between rural and urban areas. This classification was updated in 2011, distinguishing rural areas if they fall outside of areas forming settlements with populations of at least 10,000 people [27, 28]. Social deprivation was measured using the 2019 English Index of Multiple Deprivation score (IMD) that is based on seven domains: Income, Employment, Education, Health, Crime, Barriers to Housing & Services and Living Environment [27, 29]. Data are presented as quintiles of the deprivation score to prevent disclosure of patient location. Quintile 1 represents the least deprived areas and quintile 5 the most deprived areas [27]. The risk factors included the conditions asplenia or dysfunction of the spleen, chronic respiratory disease, chronic heart disease, chronic kidney disease, chronic liver disease, diabetes, immunocompromising diseases, cerebrospinal fluid leak and cochlear implant. These risk conditions were selected based on the “Clinical risk groups who should receive the pneumococcal immunization” from the Green Book chapter Pneumococcal Disease [9].

### Statistical analyses

The statistical analyses were, primarily, descriptive. They were conducted both for children aged 0–17 years and separately by age group (0–1, 2–4, and 5–17 years), sex, region, urbanicity, social deprivation and ethnicity. Regression analyses were also conducted both for children aged 0–17 years and separately by age group (0–1, 2–4, and 5–17 years). SAS Studio version 9.4 (SAS Institute, Inc., Cary, North Carolina) was utilized to perform all the analyses.

IRs of IPD, PP and ACP were calculated as the number of episodes of each definition in a calendar year divided by the total number of person-years (PY) among children aged 0–17 years in the database, per 100,000 PY. This was performed for each calendar year and for the following pneumococcal conjugate vaccine (PCV) periods: pre-PCV7 (2003–2005); post-PCV7 (2007–2009); early post-PCV13 (2011–2014); and late post-PCV13 (2015–2019). These time periods did not include the years 2006 and 2010, as they were the implementation periods of PCV7 and PCV13, respectively. Data were assumed to follow a Poisson distribution when calculating the 95% confidence intervals (CI). Interrupted Time Series (ITS) models were utilized to analyze time trends before any vaccine was introduced (pre-PCV7, 2003–2005), and after, at each of the post-PCV periods described above. Analyses were conducted for IPD and for both pneumonia definitions, overall and by each age stratum.

An exploratory analysis was performed to determine the effect of seasonality, by plotting monthly IRs during the follow-up period. The underlying trend, seasonal patterns, and outliers of time-series data points were identified using scatterplots. Generalized linear models (GLM) with Poisson distribution and a log link function (population in log PY per 100,000 as an offset for the denominator) were used for model estimation. Adjustments were made to control for seasonality using annual trends within periods. Incidence rate ratios (IRRs) with 95% CI were reported for each of the three post-PCV periods’ IRs compared to the pre-PCV7 reference period (2003–2005) before any PCV was introduced in the UK.

## Results

A total of 1,500,686 children aged 0–17 years were followed up from 2003 to 2019, providing a total of 7,435,373.4 PY at risk. Table 1 summarizes the characteristics of the study population at their inclusion in the study. The median age was 4.0 years (interquartile range, IQR 0.0–11.0) and 51.7% of the children were male. Children aged 5–17 years represented the largest group comprising 48.6% of the children. A majority of children lived in urban areas (87.6% versus 12.4% in rural areas), and in London (15.4%), the North West (15.0%), and the South East Coast (14.8%). Regarding social deprivation, the children in the study population were almost evenly distributed across quintiles, although a slightly higher proportion resided in the quintile representing the least deprived areas (quintile 1). Only 6.8% of the children had a history of any of the selected at-risk medical conditions. There was a high percentage of missing information for ethnicity (75.1% not stated/missing/inconsistent), so IRs by ethnic group were not reported.

**Table 1:** Baseline characteristics of the study population (*n* = 1,500,686) from 2003 to 2019

	N	%^*^
**Population by year**		
2003	527,885	–
2004	579,904	–
2005	627,223	–
2006	668,922	–
2007	707,332	–
2008	745,655	–
2009	766,870	–
2010	790,398	–
2011	790,747	–
2012	784,472	–
2013	772,412	–
2014	699,879	–
2015	585,480	–
2016	430,976	–
2017	341,078	–
2018	282,628	–
2019	243,745	–
**Age (years)**		
Minimum, maximum	0.0, 17.0	–
Mean, standard deviation	5.7, 5.7	–
Median (lower quartile, upper quartile)	4.0 (0.0–11.0)	–
**Age group**		
0–1 years	555,367	37.0
2–4 years	215,630	14.4
5–17 years	729,689	48.6
**Sex**		
Male	775,231	51.7
Female	725,455	48.3
**Geographic region**		
North East	29,145	1.9
North West	224,670	15.0
Yorkshire & The Humber	52,502	3.5
East Midlands	45,708	3.0
West Midlands	173,151	11.5
East of England	154,621	10.3
South West	175,419	11.7
South Central	191,935	12.8
London	231,471	15.4
South East Coast	222,064	14.8
**Urbanicity**		
Urban	1,314,679	87.6
Rural	186,007	12.4
**Social deprivation (IMD Score)**		
Quintile 1 (least deprived)	322,501	21.5
Quintile 2	293,616	19.6
Quintile 3	297,302	19.8
Quintile 4	289,738	19.3
Quintile 5 (most deprived)	295,851	19.7
Missing	1678	0.1
**Ethnicity**		
White	270,010	18.0
South Asian	43,354	2.9
Black	27,871	1.9
Other	14,403	1.0
Mixed	18,183	1.2
Not stated / Missing / Inconsistent	1,126,865	75.1
**Risk factors**		
No history of any risk medical condition	1,398,469	93.2
History of any risk medical condition^**^	102,217	6.8
Asplenia or dysfunction of the spleen	810	0.1
Chronic respiratory disease	92,424	6.2
Chronic heart disease	3806	0.3
Chronic kidney disease	585	0.0
Chronic liver disease	162	0.0
Diabetes	1946	0.1
Immunocompromising diseases	10,579	0.7
Cerebrospinal fluid leak	38	0.0
Cochlear implant	134	0.0

*Some totals sum to more or less than 100% due to rounding. ** The sum of each of the risk medical conditions does not match the total number of any risk medical condition because a patient could have more than one medical condition. IMD: Index of Multiple Deprivation; N: number

### Incidence of IPD, PP and ACP

From 2003 to 2019, 170 IPD episodes were observed in children aged ≤17 years, IR 2.29 (95% CI 1.96–2.66) per 100,000 PY. The youngest children (aged 0–1 years) had five-fold higher IPD IRs [15.52 (95% CI 12.64–18.86)] compared to children aged 2–4 years [2.86 (95% CI 2.01–3.96)] and 5–17 years [0.60 (95% CI 0.41–0.84)], per 100,000 PY (Table 2).

**Table 2:** IPD, PP and ACP IRs by patient characteristics at the time of episode (2003–2019)

	IPD		PP		ACP	
	N episodes	Rate per 100,000 PY (95% CI)	N episodes	Rate per 100,000 PY (95% CI)	N episodes	Rate per 100,000 PY (95% CI)
**All individuals**	170	2.29 (1.96–2.66)	769	10.34 (9.62–11.10)	12,142	163.37 (160.47–166.30)
**Age groups**						
0–1 years	101	15.52 (12.64–18.86)	195	29.97 (25.91–34.48)	3571	549.45 (531.57–567.77)
2–4 years	36	2.86 (2.01–3.96)	265	21.08 (18.62–23.78)	4358	346.95 (336.73–357.41)
5–17 years	33	0.60 (0.41–0.84)	309	5.59 (4.98–6.25)	4213	76.24 (73.95–78.57)
**Sex**						
Male	89	2.31 (1.86–2.85)	433	11.26 (10.22–12.37)	6543	170.22 (166.12–174.40)
Female	81	2.26 (1.79–2.80)	336	9.36 (8.39–10.42)	5599	156.02 (151.96–160.17)
**Geographic region**						
North East	7	4.47 (1.80–9.22)	15	9.59 (5.37–15.81)	292	186.73 (165.93–209.43)
North West	19	1.55 (0.93–2.42)	68	5.56 (4.31–7.04)	1881	153.75 (146.88–160.86)
Yorkshire & The Humber	8	3.17 (1.37–6.24)	38	15.05 (10.65–20.66)	399	158.13 (142.99–174.43)
East Midlands	11	5.73 (2.86–10.26)	7	3.65 (1.47–7.51)	471	245.56 (223.88–268.77)
West Midlands	11	1.21 (0.61–2.17)	70	7.73 (6.02–9.76)	1557	171.90 (163.47–180.66)
East of England	15	1.99 (1.11–3.28)	74	9.81 (7.70–12.32)	1042	138.19 (129.93–146.85)
South West	27	3.13 (2.07–4.56)	126	14.63 (12.18–17.41)	1338	155.36 (147.15–163.92)
South Central	33	3.33 (2.29–4.68)	119	12.02 (9.96–14.39)	1932	195.26 (186.65–204.16)
London	16	1.71 (0.98–2.78)	123	13.17 (10.95–15.71)	1648	176.52 (168.10–185.26)
South East Coast	23	1.97 (1.25–2.96)	129	11.08 (9.25–13.16)	1582	135.87 (129.26–142.73)
**Urbanicity**						
Urban	143	2.20 (1.86–2.60)	645	9.94 (9.19–10.74)	10,732	165.47 (162.36–168.63)
Rural	27	2.85 (1.88–4.15)	124	13.10 (10.89–15.61)	1410	148.96 (141.28–156.94)
**Social deprivation (IMD Score)**^**a**^						
Quintile 1 (least deprived)	40	2.26 (1.61–3.07)	227	12.80 (11.19–14.58)	2657	149.90 (144.25–155.71)
Quintile 2	28	1.87 (1.24–2.71)	134	8.96 (7.51–10.62)	2304	154.19 (147.95–160.61)
Quintile 3	41	2.81 (2.01–3.81)	182	12.46 (10.71–14.40)	2429	166.31 (159.76–173.05)
Quintile 4	30	2.21 (1.49–3.16)	126	9.30 (7.75–11.07)	2337	172.59 (165.66–179.73)
Quintile 5 (most deprived)	31	2.30 (1.57–3.27)	100	7.43 (6.05–9.04)	2404	178.73 (171.66–186.02)

^a^Missing values not reported. *CI* Confidence Interval, *IMD* Index of Multiple Deprivation, *IPD* Invasive Pneumococcal Disease, *N* Number, *PY* Person-Years

Results by IPD manifestation throughout the study period are presented in Supplementary Table 2. Pneumococcal meningitis had the highest IRs in the overall population [1.48 (95% CI 1.22–1.78) per 100,000 PY] and across all age groups over the study period. In contrast, the lowest incidence was for bacteremic pneumonia [IR 0.22 (95% CI 0.12–0.35) per 100,000 PY] and other IPD (IR not reported, < 5 episodes). The differences by age groups were large in pneumococcal meningitis, with IRs of 11.37 (95% CI 8.93–14.28) for children aged 0–1 years compared to 2–4 years [1.67 (95% CI 1.03–2.55)], and 5–17 years [0.27 (95% CI 0.15–0.45)] per 100,000 PY.

**Fig. 1:**
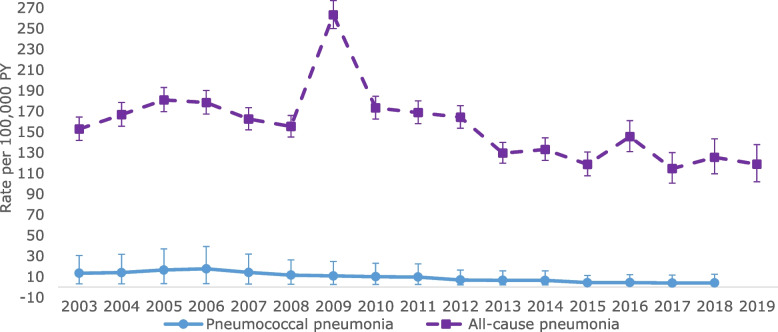
PP and ACP IRs (per 100,000 PY) by study year (2003–2019). ACP: all-cause pneumonia; PP: pneumococcal pneumonia; PY: Person-Years

From 2003 to 2019, the PP IR was 10.34 (95% CI 9.62–11.10) and the ACP IR was 163.37 (95% CI 160.47–166.30) per 100,000 PY (Table 2). For ACP, a peak was observed in 2009 driven by the Read diagnosis code *11849: Other specified pneumonia or influenza* in primary care (Fig. 1 and Supplementary Fig. 3). By age group, both PP and ACP IRs were highest in children aged < 5 years and were as follows: for PP 0 to 1 year: 29.97 (95% CI 25.91–34.48); 2–4 years: 21.08 (95% CI 18.62–23.78); 5–17 years: 5.59 (95% CI 4.98–6.25); for ACP 0 to 1 year: 549.45 (95% CI 531.57–567.77); 2–4 years: 346.95 (95% CI 336.73–357.41); 5–17 years: 76.24 (95% CI 73.95–78.57) (all per 100,000 PY) (Table 2).

Differences were observed across the different geographic regions. The highest IPD and ACP IRs were observed in the East Midlands, 5.73 (95% CI 2.86–10.26) and 245.56 (95% CI 223.88–268.77) per 100,000 PY, respectively. For PP, the highest IRs were 15.05 (95% CI 10.65–20.66) per 100,000 PY in Yorkshire & The Humber. For ACP, IRs were lowest in children living in the least deprived areas and increased with increasing deprivation, with the highest IRs in the most deprived areas. IPD, PP and ACP IRs by patient characteristics, including sex, urbanicity, geographic region, and social deprivation are shown in Table 2.

### Incidence by PCV period

The overall crude-incidence monthly ratios within period linear trends are presented in the Supplementary appendix for the three definitions; IPD (Supplementary Fig. 1), PP (Supplementary Fig. 2) and ACP (Supplementary Fig. 3). A decrease was observed across the study period (2003–2019) for IPD, PP and ACP overall, except for the post-PCV7 period (2007–2009) in ACP, and the early post-PCV13 (2011–2014) in IPD and ACP.

Table 3 summarizes the change in IRs across the four PCV periods, in addition to IRRs for each period (versus pre-PCV7 period; adjusting for seasonal variations and the ‘within PCV period annual trend’). A significant decrease in IRs was observed from the pre-PCV7 (2003–2005, before any PCV was introduced in the UK) to the late post-PCV period (2015–2019) for IPD, PP and ACP. IPD IRs halved from 3.28 (95% CI 2.42–4.33) to 1.41 (95% CI 0.80–2.29) per 100,000 PY: IRR 0.28 (95% CI 0.09–0.90), *p*-value 0.033. PP IRs decreased from 14.65 (95% CI 12.77–16.72) to 3.87 (95% CI 2.81–5.20) per 100,000 PY: IRR 0.19 (95% CI 0.09–0.38), p-value < 0.001. ACP IRs decreased from 167.28 (95% CI 160.78–173.96) to 124.96 (95% CI 118.54–131.63) per 100,000 PY: IRR 0.77 (95% CI 0.66–0.88), p-value < 0.001.

**Table 3:** IRs and IRRs before and after the introduction of PCV7 and PCV13

	N episodes	IRs per 100,000 PY (95% CI)	IRR (95% CI) vs. reference period (pre-PCV7)^a^	p-value
**Pre-PCV7 (2003–2005)**				
IPD	49	3.28 (2.42–4.33)	1	–
PP	219	14.65 (12.77–16.72)	1	–
ACP	2500	167.28(160.78–173.96)	1	–
**Post-PCV7 (2007–2009)**				
IPD	40	2.38 (1.70–3.25)	0.41 (0.17–1.01)	0.052
PP	204	12.16 (10.55–13.95)	0.66 (0.44–0.99)	0.046
ACP	3253	193.95(187.35–200.74)	1.00 (0.88–1.12)	0.945
**Early post-PCV13 (2011–2014)**				
IPD	34	1.68 (1.16–2.34)	0.52 (0.22–1.25)	0.143
PP	151	7.45 (6.31–8.73)	0.47 (0.30–0.72)	0.001
ACP	3041	150.00 (144.72–155.43)	0.98 (0.87–1.10)	0.732
**Late post-PCV13 (2015–2019)**				
IPD	16	1.41 (0.80–2.29)	0.28 (0.09–0.90)	0.033
PP	44	3.87 (2.81–5.20)	0.19 (0.09–0.38)	< 0.001
ACP	1420	124.96 (118.54–131.63)	0.77 (0.66–0.88)	< 0.001

^a ^Pre-PCV7 (2004–2005). *ACP* All-cause Pneumonia, *CI* Confidence Interval, *IPD* Invasive Pneumococcal Disease, *IR* Incidence Rate, *IRR* Incidence Rate Ratio, *N* Number, *PCV* Pneumococcal Conjugate Vaccine, *PP* Pneumococcal Pneumonia, *PY* Person-Years

Results by age group for IPD, PP and ACP are presented in Supplementary Tables 3 and 4. For the three definitions, IRs were highest in the youngest children (< 2 years) across the four PCV periods. The decrease in IPD IRs (IRR for the late post-PCV13 period compared to the pre-PCV7 period) was not significant when stratified by age group. For PP, the IRRs for the late post-PCV13 period compared to pre-PCV7, showed a significant decrease in all three age groups. Regarding ACP, the IRR for the late post-PCV13 period compared to the pre-PCV7 period was significant in both children aged 0–1 years (IRR 0.63, 95% CI 0.47–0.85, *p*-value 0.003) and children aged 2–4 years, (IRR 0.71, 95% CI 0.57–0.89, *p*-value 0.003), but not for those aged 5–17 years (IRR 0.87, 95% CI 0.68–1.10, p-value 0.233).

## Discussion

Pneumococcal infections and IPD are major causes of communicable disease morbidity and mortality in Europe and globally in young children [1]. This large retrospective cohort study reports the most recent IPD and PP and ACP IRs and trends across PCV periods from 2003 to 2019 in children aged ≤17 years in England. Across the study period (2003–2019), the youngest children (0–1 years) had the highest IRs. Our study shows a reduction in IRs across PCV periods in children aged ≤17 years from pre-PCV7 (2003–2005, before any PCV was introduced in the UK) to late post-PCV13 (2015–2019); IPD IRs halved from 3.28 (95% CI 2.42–4.33) to 1.41 (95% CI 0.80–2.29); PP IRs decreased from 14.65 (95% CI 12.77–16.72) to 3.87 (95% CI 2.81–5.20); and ACP IRs decreased from 167.28 (95% CI 160.78–173.96) to 124.96 (95% CI 118.54–131.63) (all presented per 100,000 PY). The decrease in IRs in the late post-PCV13 period versus the pre-PCV7 period, was significant for all three definitions: IPD: IRR 0.28 (95% CI 0.09–0.90), *p*-value 0.033; PP: IRR 0.19 (95% CI 0.09–0.38), p-value < 0.001; and ACP: IRR 0.77 (95% CI 0.66–0.88), p-value < 0.001.

The decrease in IPD IRs observed in our analysis has been shown in previous studies conducted in England and Wales [6, 12, 30]. In children aged < 2 years, Waight PA., et al. using surveillance data, reported an IPD incidence decrease from 22.22 in 2008–2010 to 12.03 in 2013–2014 per 100,000 PY, IRR 0.54 (95% CI 0.42–0.69) [12]. In Ladhani SN., et al., also using surveillance data, IRs in children aged < 2 years decreased from 49.00 in 2000–2006, to 13.90 in 2016–2017 per 100,000 PY, giving an IRR of 0.28 (95% CI 0.23–0.35) [6]. In our study, the late post-PCV13 period (2015–2019) IR was 10.72 (95% CI 5.14–19.72) per 100,000 PY for children aged < 2 years. Oligbu G., et al. was focused on pneumococcal meningitis in children aged < 5 years, and observed in surveillance data a reduction in IRs from 3.10 in 2008–2010 to 1.22 in 2015–2016 per 100,000 PY, giving an IRR of 0.39 (95% CI 0.25–0.63) [30]. In our study, meningitis was the most common IPD manifestation across all age groups, with a similar IR of 1.48 (95% CI 1.22–1.78) across the study period in children aged 0–17 years. Also, using surveillance data from England, Kent A., et al., in infants aged < 1 year in 2013–2016, reported an IPD incidence of 19 cases per 100,000 infants, which was not dissimilar to our study estimate of 15.52 (95% CI 12.64–18.86) per 100,000 PY in children 0–1 year old in 2003–2019 [31].

Comparisons with studies using surveillance data should be interpreted with caution. Prior studies have demonstrated the importance of comparing surveillance data to data from other sources, to better interpret observed trends [32]. Only one previous study has reported IPD IRs using administrative health data in England. Thorrington D., et al., using the HES database to identify IPD and PP episodes from 2004 to 2015 [13], reported a reduction of 72% in IPD [IRR 0.28 (95% CI 0.25–0.32)] and 80% in PP [IRR 0.20 (95% CI 0.15–0.26)] in children aged < 2 years. In our study we also observed a reduction of PP IRs in children < 2 years across pre-PCV7 (2003–2005) and late post-PCV13 (2015–2019), from 42.68 (95% CI 31.97–55.83) to 12.87 (95% CI 6.65–22.47) per 100,000 PY, IRR 00.26 (95% CI 0.06–1.12) *p*-value 0.070.

To our knowledge, there are no recent studies reporting IRs for ACP identified in children both in primary care and in hospital in England. Using primary care data from the IMS Disease Analyser database in the UK, Lau WCY., et al. identified children aged 0–9 years with ACP from 2002 to 2012. After the introduction of PCV7, there was no immediate reduction in the incidence of pneumonia in children aged under 2 years (IRR 1.04, 95% CI 0.86–1.24) [14]. However, the incidence of pneumonia then declined gradually over the post-PCV7 period (IRR 0.98, 95% CI 0.97–0.99). Similarly, there was a gradual decline in the trend in pneumonia incidence during the post-PCV7 period in children aged 2 to 4 years (IRR 0.99, 95% CI 0.98–0.99) and 5 to 9 years (IRR 0.99, 95% CI 0.99–1.00). Following the introduction of PCV13, no immediate change in pneumonia incidence was observed in children aged under 2 years (IRR 1.09, 95% CI 0.81–1.49), 2 to 4 years (IRR 0.86, 95% CI 0.68–1.07), and 5 to 9 years (IRR 0.92, 95% CI 0.73–1.15). In our study we also reported declines in ACP IRs in children ≤17 years in the post-PCV period, and in children aged 2–4 years: from 376.94 (95% CI 352.85–402.23) in the pre-PCV7 to 285.21 (95% CI 261.74–310.21) in the late post-PCV13 per 100,000 PY, IRR 0.71 (95% CI 0.57–0.89), *p*-value 0.003. Another study, also conducted in the CPRD database from 2002 to 2012, by Sun X., et al., reported a reduction in clinically diagnosed ACP IRs in children under 15 years of age [15]. A further study, using HES hospital data from England during 2001–2014 conducted by Saxena S., et al., found a significant decrease in pneumonia admissions in all age groups immediately following PCV7 introduction [16]. The largest drop was seen in children aged < 2 years [rate ratio (RR) 0.80; 95% CI 0.73–0.88] and 5–9 years (RR 0.80; 95% CI 0.72–0.89) but trends in pneumonia admissions began to rise again in the PCV7 era for all age groups [16].

In our study we observed a peak in 2009 of ACP in primary care (Fig. 1) which is likely the reflection of the 2009 H1N1 influenza pandemic [33]. Looking at the code list frequency, this peak was driven by Read diagnosis code 11849: Other specified pneumonia or influenza in primary care. This peak was not observed in Lau WCY., et al., but this is likely explained by the difference in code lists. Lau WCY. et al., included code H062: acute low respiratory tract infection, which was the most frequent code, thereby likely attenuating the effect of Read diagnosis code 11849 [14].

By social deprivation, the only clear trend in IRs was observed in ACP. ACP IRs increased with increasing deprivation with children living in the most deprived areas (Quintile 5) having the highest ACP IRs. This trend is in alignment with a previous study conducted in the West Midlands Health Region of England using HES data from April 1990 to March 1995 that reported pneumonia hospital admissions were significantly associated with deprivation [34]. A further study in 198,572 newborns during 2005–2010 in Canada conducted in pediatric respiratory diseases found a higher concentration of ED visits and hospitalizations for pediatric respiratory diseases in the most deprived groups [35].

The main strength of this study is the size of the study population and representativeness. Previous studies have demonstrated that CPRD-Gold is representative of the UK general population in terms of age, sex and ethnicity [24], and HES APC include all admissions to NHS hospitals in England [23]. Another strength of our study is the analysis of IR trends across PCV periods. We excluded the years of implementation of PCV7 and PCV13, 2006 and 2010, respectively, to allow for a better estimation of the impact of PCVs. Another added value of this study was the inclusion of more years in the post-PCV13 period, allowing for observation of the consistency of the effect of vaccination. It also allowed for the comparison of the effect in the short term with other studies. Previous studies conducted in the UK, included up to 2017 for IPD (Ladhani SN., et al. [6]), up to 2015 for PP (Thorrington D., et al. [13]) and up to 2017 for ACP (Sun X., et al. [15]).

A further strength lies in the choice of analysis method. The ITS design offers a robust quasi-experimental alternative for evaluating treatment effects when data are available for multiple time points in both the pre-intervention and post-intervention periods. The advantage of ITS is the ability to control for secular trends and seasonality in population-level data.

There were, however, a number of limitations to this study. First, there was a reduction in the size of the study population in CPRD-Gold from 2015 onwards. This is explained by the migration of GP practices from one GP software to another [36]. Despite this reduction in study population size, CPRD-Gold continues to be representative of the UK population [24]. Second, the true perinatal morbidity of infants is likely an underestimate in this study due to the requirement of a minimum look-back period for the estimation of morbidity for children less than 12 months old.

This was a descriptive study with the aim of estimating the IPD, PP and ACP incidence rates, so no adjustments at the time of the episode for covariates or vaccination status were included in our ITS models. Results are therefore presented as crude rates.

Finally, lab results, medical charts and serotype distribution were not available to verify coding or diagnoses. This may have led to underestimation of the true incidence of IPD and pneumonia. Despite, *S. pneumoniae* being the most common cause of community acquired bacterial pneumonia [1] we identified few PP episodes. In clinical practice, especially in the primary care setting, initial pneumonia diagnosis is typically made on clinical judgment without radiological confirmation or knowledge of the causative organism [37]. Therefore, a wider pneumonia definition, ACP, was also included to capture all pneumonia episodes caused by any organisms (bacterial or viral), to provide a further estimate of the burden of pneumococcal disease. This approach has been used previously [13, 38–40]. Lack of information on causative pneumococcal serotypes for IPD and PP in CPRD or HES also meant that it was not possible to explore changing serotype distribution across PCV periods. An understanding of pneumococcal serotype distribution, particularly of prevalent and emerging serotypes, will be essential when considering the potential value of novel PCVs to reduce the burden of pneumococcal disease. Furthermore, studies will also be needed to determine the impact of SARS-CoV-2 on the immunization schedule of the novel PCVs.

To minimize misclassification bias, we carefully and thoroughly evaluated pneumococcal-specific and unspecified diagnoses used to identify pneumococcal-related infections as well as rules for defining episodes. In doing so, we referred to the literature and clinical experts as appropriate. While these steps would not have prevented coding errors or omissions, it did reduce the risk of misclassification due to lack of specificity or sensitivity of the diagnosis codes used to identify pneumococcal-related infections, or the episode definitions that are not reflective of the typical duration of illness.

## Conclusions

The clinical burden of IPD, PP and ACP declined in children aged 0–17 years between 2003 and 2019, especially in the post-PCV13 period in England. This study highlights the importance of PCVs in reducing the burden of pneumococcal disease and ACP in children in England.

## Supplementary Information

**Additional file 1: Supplementary Table 1.** IPD, PP and ACP diagnosis code lists, **Supplementary Table 2.** IPD IRs by age and manifestations at the time of episode (2003-2019), **Supplementary Table 3.** Overall IPD, PP and ACP IRs by study year (2003-2019), **Supplementary Fig. 1.** Incidence Monthly Ratio of IPD from 2003 to 2019, **Supplementary Fig. 2.** Incidence Monthly Ratio of PP from 2003 to 2019, **Supplementary Fig. 3.** Incidence Monthly Ratio of ACP from 2003 to 2019, **Supplementary Table 4.** IRs and IRRs before and after the introduction of PCV7 and PCV13 by age groups.

## Data Availability

The data used for this study were obtained from the CPRD and the HES APC. The datasets generated during and/or analyzed during the current study are not publicly available.

## Abbreviations

ACP: All-cause pneumonia
CI: Confidence interval
CORE: Center for Observational and Real-World Evidence
CPRD: Clinical Practice Research Datalink
GBD: Global Burden of Diseases, Injuries, and Risk Factors
GLM: Generalized Linear Models
HES APC: Hospital Episode Statistics Admitted Patient Care
ICD-10: International Statistical Classification of Diseases and Related Health Problems 10^th^ revision
IMD: Indices of Multiple Deprivation
IPD: Invasive pneumococcal disease
IQR: Interquartile range
IR: Incidence rate
IRR: Incidence rate ratio
ISAC: Independent Scientific Advisory Committee
ITS: Interrupted Time Series
JCVI: Joint Committee on Vaccination and Immunisation
LRI: Lower respiratory infections
NHS: National Health Service
PCV: Pneumococcal conjugate vaccine
PCV13: 13-valent pneumococcal conjugate vaccine
PCV15: 15-valent pneumococcal conjugate vaccine
PCV20: 20-valent pneumococcal conjugate vaccine
PCV7: 7-valent pneumococcal conjugate vaccine
PP: Pneumococcal pneumonia
PY: Person-years
RDAI: Real-world Data Analytics and Innovation
RR: Rate ratio
*S. pneumoniae*: *Streptococcus pneumoniae*
TESSy: The European Surveillance System
UTS: Up-to-standard
VAD: Value, Access and Devolved nations
