# Supplementary material for: Incidence of pneumococcal disease from 2003 to 2019 in children ≤17 years in England
Source: Pneumonia (Nathan). 2023 Jan 23;15:2. doi: 10.1186/s41479-022-00103-3 (PMC9868000; doi:10.1186/s41479-022-00103-3)
Supplement: Supplementary file 1 — Additional file 1: Supplementary Table 1. IPD, PP and ACP diagnosis code lists, Supplementary Table 2. IPD IRs by age and manifestations at the time of episode (2003-2019), Supplementary Table 3. Overall IPD, PP and ACP IRs by study year (2003-2019), Supplementary Fig. 1. Incidence Monthly Ratio of IPD from 2003 to 2019, Supplementary Fig. 2. Incidence Monthly Ratio of PP from 2003 to 2019, Supplementary Fig. 3. Incidence Monthly Ratio of ACP from 2003 to 2019, Supplementary Table 4. IRs and IRRs before and after the introduction of PCV7 and PCV13 by age groups. [file 41479_2022_103_MOESM1_ESM.docx]

# Supplementary information

**Supplementary table 1** IPD, PP and ACP diagnosis code lists

| **Definition** | **Read diagnosis codes** | **ICD-10 diagnosis codes** |
| --- | --- | --- |
| **IPD** |  |  |
| Pneumococcal bacteremia/ septicemia | - | A40.3, A40.9+B95.3 |
| Pneumococcal meningitis | - | G00.1, G00.2+B95.3 |
| Pneumococcal bacteremic pneumonia | - | J13+A40.3, J13+A40.9, J13+A41.9, J13+A49.9, J86.0+B95.3, J86.9+B95.3, J85.1+B95.3 |
| Other IPD | - | I30.1+B95.3, I33.0+B95.3, I33.9+B95.3, K65.0+B95.3, K65.8+B95.3, K65.9+B95.3, M00.1, M86.1+B95.3, M86.2+B95.3, M86.9+B95.3 |
| **PP** | AyuK300, A3BX400, H223.00, H21..00, H21..11 | J13 |
| **ACP** | 14B2.00, 43eG.00, 43eH.00, 43n1.00, 43n7.00, 4JRC.00, 4JUK.00, A022200, A116.00, A203.00, A204.00, A205.00, A3A4000, A3BXA00, A3BXB00, A3By400, A521.00, A54x400, A551.00, A730.00, A785000, A789300, A789311, A789900, AB24.11, AB40500, AB41500, AD04.00, AyuK900, AyuKA00, H060A00, H2...00, H20..00, H20..11, H200.00, H201.00, H202.00, H203.00, H20y.00, H20z.00, H21..00, H21..11, H22..00, H22..11, H220.00, H221.00, H222.00, H222.11, H223.00, H223000, H224.00, H22y.00, H22y000, H22y011, H22y100, H22y200, H22yX00, H22yz00, H22z.00, H23..00, H23..11, H230.00, H231.00, H232.00, H233.00, H23z.00, H24..00, H240.00, H241.00, H242.00, H243.00, H243.11, H244.00, H246.00, H247.00, H247000, H247100, H247z00, H24y.00, H24y000, H24y100, H24y200, H24y300, H24y400, H24y500, H24y600, H24y700, H24yz00, H24z.00, H25..00, H25..11; H26..00; H260.00; H261.00; H262.00; H263.00; H270.00; H270.11; H270000; H270100; H270z00; H28..00; H2B..00; H2C..00; H2y..00; H2z..00; H35..11; H357.00; H35z.00; H35z100; H35zz00; H460.00; H460100; H460z00; H47..00; H47..11; H470.00; H470.11; H470000; H470100; H470200; H470211; H470300; H470311; H470312; H470z00; H471.00; H471000; H471z00; H472.00; H47y.00; H47yz00; H47z.00; H4y0000; H530200; H530300; H540000; H540100; H563300; H564.00; H564.11; H56y000; H56y100; H571.00; Hyu0800; Hyu0A00; Hyu0B00; Hyu0C00; Hyu0D00; Hyu0G00; Hyu0H00; Hyu4300; Hyu4700; Q310.00; Q310000; Q310100; Q310200; Q310300; Q310400; Q310500; Q310600; Q310y00; Q310z00; Q311z11; SP13100; AyuK300; A3BX400 | A22.1, A37.9, A48.1, B25.0,B44.0, J10.0, J11.0, J12.0, J12.1, J12.2, J12.3, J12.8, J12.9, J13, J14, J15.0, J15.1, J15.2, J15.3, J15.4, J15.5, J15.6, J15.7, J15.8, J15.9, J16.0, J16.8, J17.0, J17.1, J17.2, J17.3, J17.8, J18.0, J18.1, J18.2, J18.8, J18.9 |

ICD-10: International Statistical Classification of Diseases and Related Health Problems 10th revision; IPD: Invasive Pneumococcal Disease.

**Supplementary table 2** IPD IRs by age and manifestations at the time of episode (2003-2019)

|  | **N episodes** | **Rate per 100000 PY (95% CI)** |
| --- | --- | --- |
| **All individuals** |  |  |
| Bacteremia/septicemia | 45 | 0.61 (0.44-0.81) |
| Meningitis | 110 | 1.48 (1.22-1.78) |
| Bacteremic pneumonia | 16 | 0.22 (0.12-0.35) |
| Other IPD | NR* | - |
| **0-1 years** |  |  |
| Bacteremia/septicemia | 24 | 3.69 (2.36-5.49) |
| Meningitis | 74 | 11.37 (8.93-14.28) |
| Bacteremic pneumonia | NR* | - |
| Other IPD | NR* | - |
| **2-4 years** |  |  |
| Bacteremia/septicemia | 10 | 0.80 (0.38-1.46) |
| Meningitis | 21 | 1.67 (1.03-2.55) |
| Bacteremic pneumonia | 5 | 0.40 (0.13-0.93) |
| Other IPD | NR* | - |
| **5-17 years** |  |  |
| Bacteremia/septicemia | 11 | 0.20 (0.10-0.36) |
| Meningitis | 15 | 0.27 (0.15-0.45) |
| Bacteremic pneumonia | 7 | 0.13 (0.05-0.26) |
| Other IPD | 0 | 0.00 (0.0-0.07) |

*Where the number of episodes was less than 5 the data are not shown, in accordance with data protection policies. CI: Confidence interval; IPD: Invasive Pneumococcal Disease; IRs: Incidence Rates; NR: Not Reported; PY: Person-Years.

**Supplementary table 3** Overall IPD, PP and ACP IRs by study year (2003-2019)

|  | **IPD** | | **PP** | | **ACP** | |
| --- | --- | --- | --- | --- | --- | --- |
|  | **N episodes** | **Rate per 100000 PY (95% CI)** | **N episodes** | **Rate per 100000 PY (95% CI)** | **N episodes** | **Rate per 100000 PY (95% CI)** |
| **2003** | 14 | 2.97  (1.63-4.99) | 63 | 13.38  (10.28-17.11) | 719 | 152.70  (141.74-164.28) |
| **2004** | 16 | 3.19  (1.82-5.18) | 70 | 13.95  (10.88-17.63) | 836 | 166.68  (155.57-178.37) |
| **2005** | 19 | 3.64  (2.19-5.68) | 86 | 16.46  (13.17-20.33) | 945 | 180.99  (169.64-192.91) |
| **2006** | 21 | 3.90  (2.42-5.96) | 95 | 17.65  (14.28-21.58) | 960 | 178.45  (167.34-190.11) |
| **2007** | 15 | 2.72  (1.52-4.49) | 78 | 14.16  (11.20-17.68) | 894 | 162.40  (151.93-173.40) |
| **2008** | 17 | 3.03  (1.76-4.84) | 65 | 11.57  (8.93-14.74) | 872 | 155.24  (145.11-165.90) |
| **2009** | 8 | 1.42  (0.61-2.79) | 61 | 10.79  (8.25-13.86) | 1487 | 263.18  (249.97-276.91) |
| **2010** | 10 | 1.79  (0.86-3.29) | 56 | 10.02  (7.57-13.01) | 968 | 173.19  (162.45-184.45) |
| **2011** | 10 | 1.83  (0.88-3.37) | 53 | 9.71  (7.28-12.71) | 920 | 168.68  (157.96-179.94) |
| **2012** | 16 | 2.97  (1.70-4.82) | 37 | 6.87  (4.83-9.46) | 884 | 164.12  (153.47-175.30) |
| **2013** | NR* | - | 33 | 6.51  (4.48-9.15) | 656 | 129.52  (119.80-139.83) |
| **2014** | NR* | - | 28 | 6.41  (4.26-9.26) | 581 | 133.02  (122.42-144.29) |
| **2015** | 9 | 2.55  (1.17-4.85) | 15 | 4.26  (2.38-7.02) | 418 | 118.62  (107.52-130.56) |
| **2016** | NR* | - | 11 | 4.30  (2.14-7.69) | 372 | 145.31  (130.92-160.85) |
| **2017** | NR* | - | 8 | 3.88  (1.68-7.65) | 236 | 114.50  (100.36-130.08) |
| **2018** | NR* | - | 7 | 4.01  (1.61-8.26) | 219 | 125.49  (109.42-143.26) |
| **2019** | NR* | - | NR* | - | 175 | 118.74  (101.80-137.70) |

*Where the number of episodes was less than 5 the data are not shown, in accordance with data protection policies. CI: Confidence interval; IPD: Invasive Pneumococcal Disease; IR: Incidence Rate; N: Number; NR: Not Reported; PY: Person-Years.

**Supplementary figure 1** Incidence Monthly Ratio of IPD from 2003 to 2019


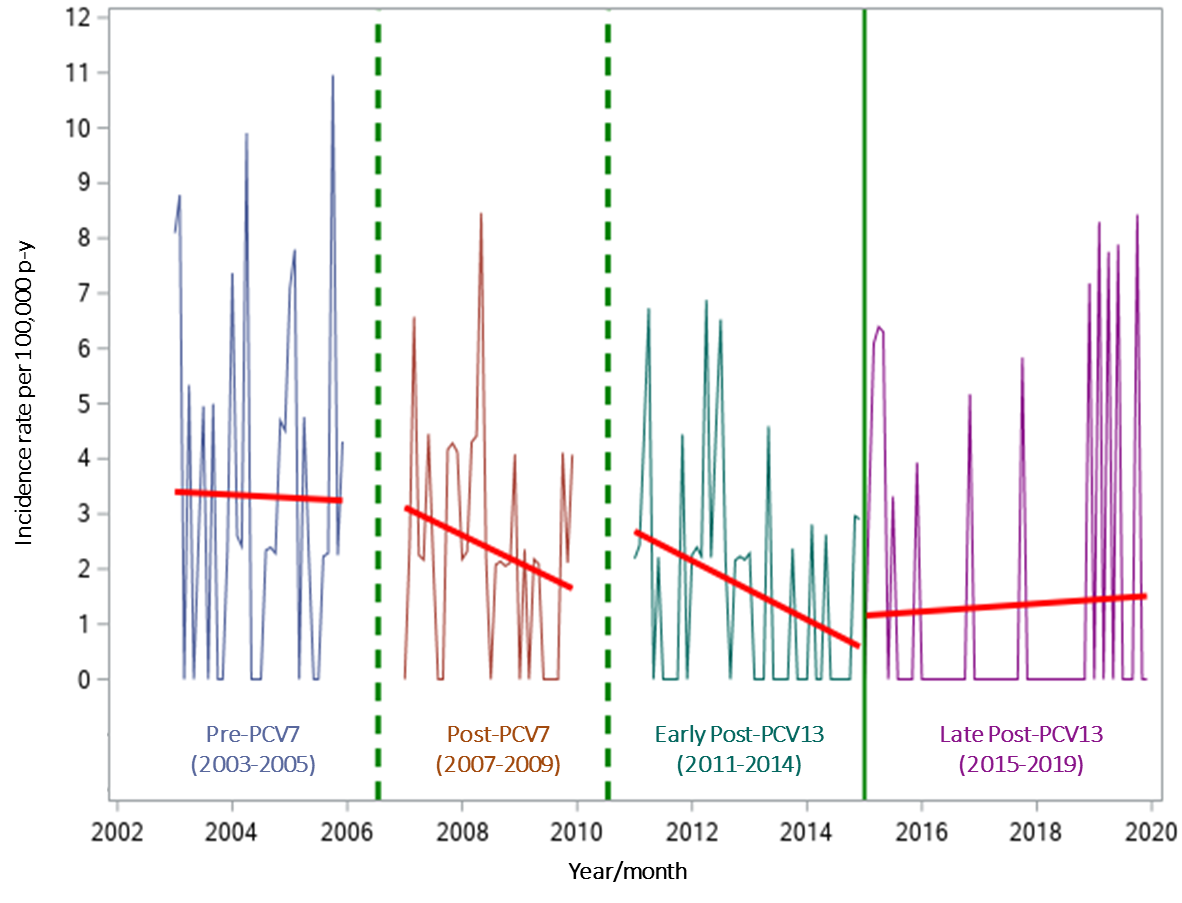


IPD: Invasive Pneumococcal Disease; PCV: Pneumococcal Conjugate Vaccine; PY: Person-Years. The dashed vertical lines correspond to the years of PCV introduction (2006 and 2010) where monthly episode rates of these years were not calculated. The solid vertical line denotes the separation of early and late post-PCV13 periods, where all years were included in the monthly episode rates.

**Supplementary figure 2** Incidence Monthly Ratio of PP from 2003 to 2019


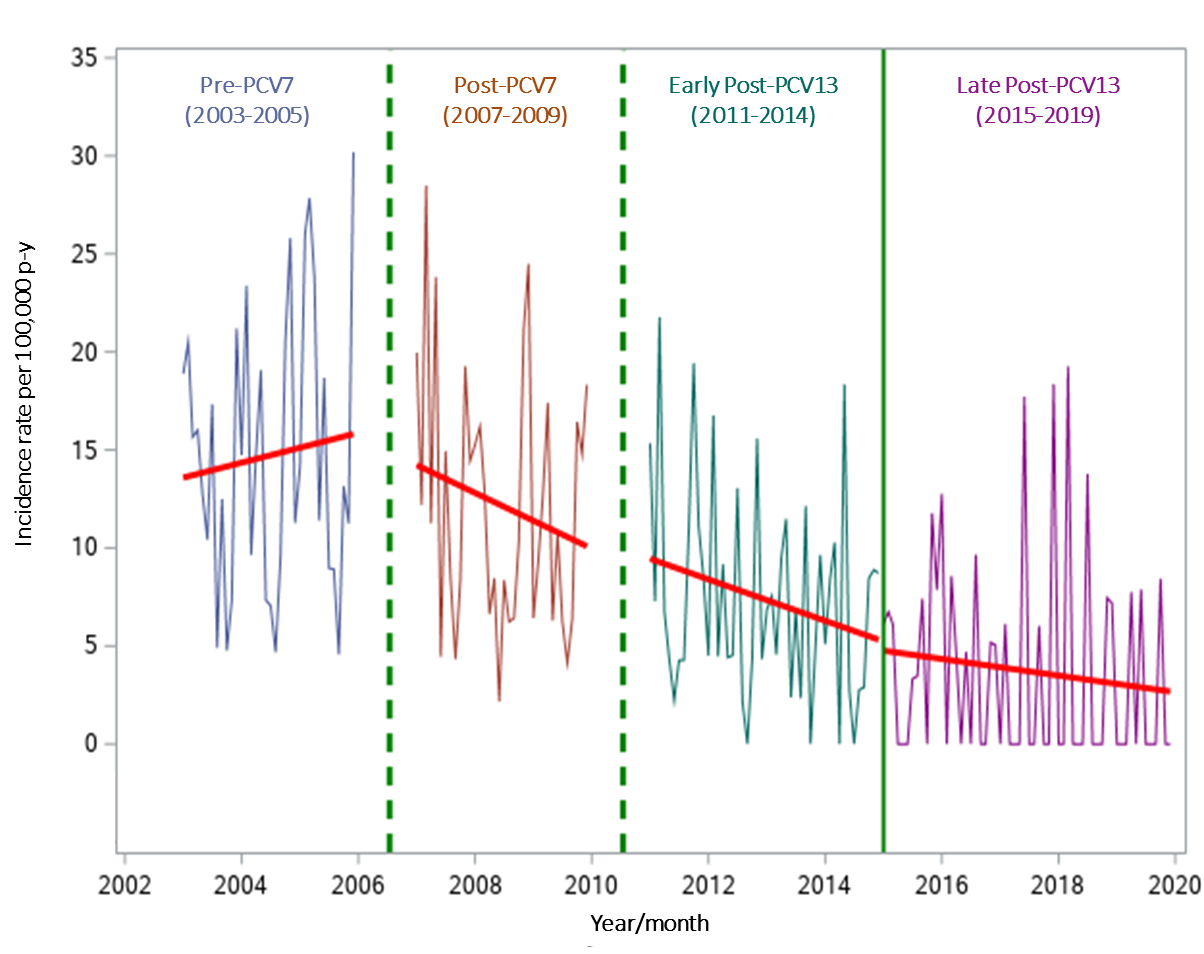


PCV: Pneumococcal Conjugate Vaccine; PY: Person-Years. The dashed vertical lines correspond to the years of PCV introduction (2006 and 2010) where monthly episode rates of these years were not calculated. The solid vertical line denotes the separation of early and late post-PCV13 periods, where all years were included in the monthly episode rates.

**Supplementary figure 3** Incidence Monthly Ratio of ACP from 2003 to 2019


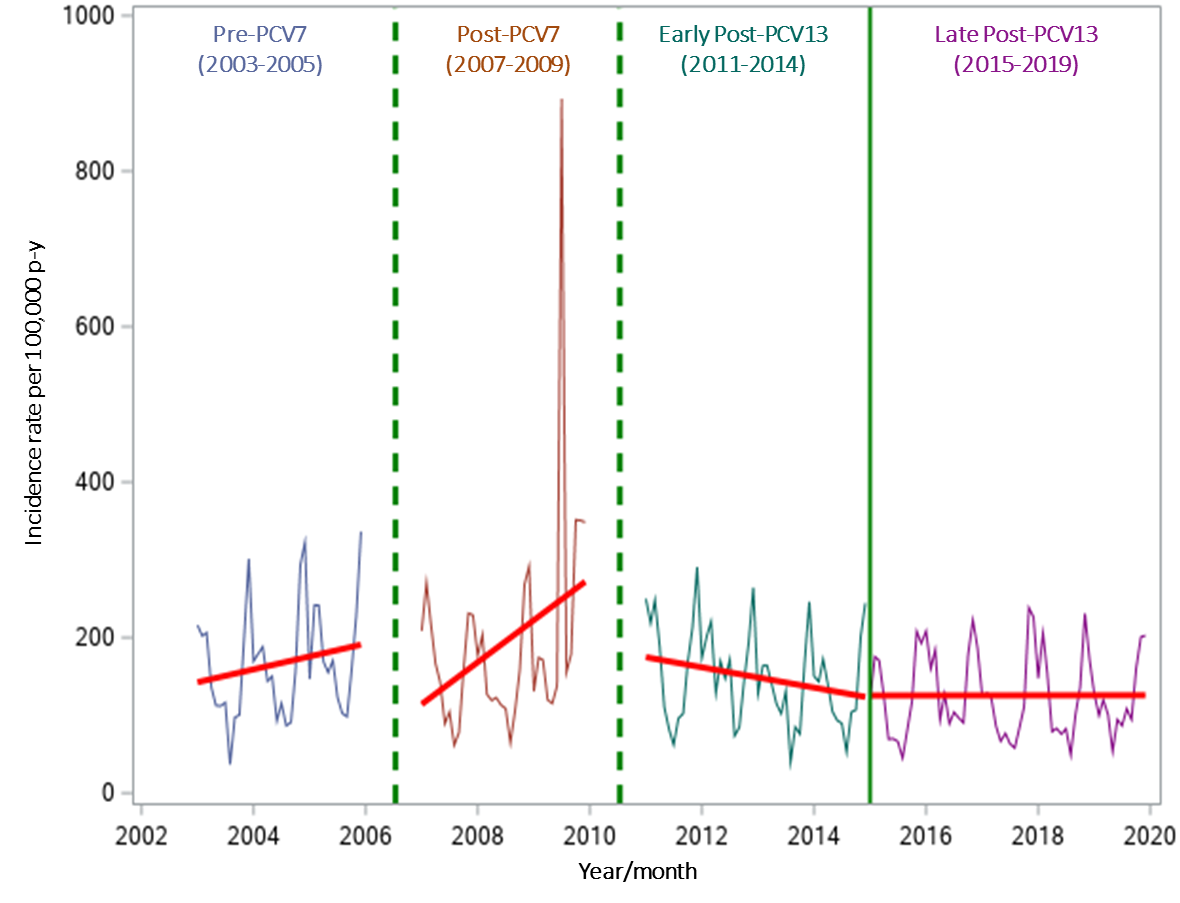


ACP: All-cause Pneumonia; PCV: Pneumococcal Conjugate Vaccine; PY: Person-Years. The dashed vertical lines correspond to the years of PCV introduction (2006 and 2010) where monthly episode rates of these years were not calculated. The solid vertical line denotes the separation of early and late post-PCV13 periods, where all years were included in the monthly episode rates.

**Supplementary table 4** IRs and IRRs before and after the introduction of PCV7 and PCV13 by age groups

|  | **N episodes** | **Rate per 100000 PY**  **(95% CI)** | **IRR (95% CI) vs. reference period (pre-PCV7)*** | **p-value** |  |
| --- | --- | --- | --- | --- | --- |
| **IPD** | | | | | |
| **Pre-PCV7 (2003-2005)** | | | | | |
| 0-1 years | 32 | 25.77 (17.63-36.38) | 1 | - |  |
| 2-4 years | 11 | 4.55 (2.27-8.14) | 1 | - |  |
| 5-17 years | 6 | 0.53 (0.20-1.16) | 1 | - |  |
| **Post-PCV7 (2007-2009)** | | | | | |
| 0-1 years | 24 | 15.79 (10.12-23.49) | 0.44 (0.13-1.43) | 0.170 |  |
| 2-4 years | 7 | 2.47 (0.99-5.10) | 0.04 (0.00-0.47) | 0.010 |  |
| 5-17 years | 9 | 0.72 (0.33-1.37) | 1.78 (0.23-13.88) | 0.582 |  |
| **Early Post-PCV13 (2011-2014)** | | | | | |
| 0-1 years | 15 | 8.17 (4.57-13.48) | 0.31 (0.08-1.12) | 0.074 |  |
| 2-4 years | 10 | 2.81 (1.35-5.16) | 0.47 (0.10-2.10) | 0.322 |  |
| 5-17 years | 9 | 0.60 (0.28-1.15) | 1.25 (0.15-10.14) | 0.835 |  |
| **Late Post-PCV13 (2015-2019)** | | | | | |
| 0-1 years | 10 | 10.72 (5.14-19.72) | 0.29 (0.06-1.33) | 0.111 |  |
| 2-4 years | NR** | - | 0.60 (0.04-8.07) | 0.700 |  |
| 5-17 years | NR** | - | 0.30 (0.01-6.15) | 0.437 |  |
| **PP** | | | | |  |
| **Pre-PCV7 (2003-2005)** | | | | |  |
| 0-1 years | 53 | 42.68 (31.97-55.83) | 1 | - |  |
| 2-4 years | 88 | 36.38 (29.18-44.82) | 1 | - |  |
| 5-17 years | 78 | 6.91 (5.46-8.62) | 1 | - |  |
| **Post-PCV7 (2007-2009)** | | | | |  |
| 0-1 years | 55 | 36.19 (27.26-47.10) | 0.71 (0.29-1.72) | 0.450 |  |
| 2-4 years | 58 | 20.50 (15.57-26.51) | 0.49 (0.24-0.99) | 0.047 |  |
| 5-17 years | 91 | 7.32 (5.89-8.99) | 0.73 (0.40-1.34) | 0.308 |  |
| **Early Post-PCV13 (2011-2014)** | | | | |  |
| 0-1 years | 44 | 23.98 (17.42-32.19) | 0.69 (0.28-1.71) | 0.427 |  |
| 2-4 years | 46 | 12.92 (9.46-17.23) | 0.28 (0.13-0.61) | 0.001 |  |
| 5-17 years | 61 | 4.10 (3.13-5.26) | 0.48 (0.25-0.94) | 0.031 |  |
| **Late Post-PCV13 (2015-2019)** | | | | |  |
| 0-1 years | 12 | 12.87 (6.65-22.47) | 0.26 (0.06-1.12) | 0.070 |  |
| 2-4 years | 19 | 9.95 (5.99-15.54) | 0.13 (0.04-0.41) | 0.001 |  |
| 5-17 years | 13 | 1.52 (0.81-2.61) | 0.24 (0.08-0.72) | 0.011 |  |
| **ACP** |  |  |  |  |  |
| **Pre-PCV7 (2003-2005)** | | | | |  |
| 0-1 years | 800 | 645.07 (601.14-691.37) | 1 | - |  |
| 2-4 years | 911 | 376.94 (352.85-402.23) | 1 | - |  |
| 5-17 years | 789 | 69.90 (65.10-74.95) | 1 | - |  |
| **Post-PCV7 (2007-2009)** | | | | |  |
| 0-1 years | 899 | 592.19 (554.11-632.20) | 0.84 (0.66-1.07) | 0.158 |  |
| 2-4 years | 1072 | 379.32 (356.95-402.73) | 0.82 (0.68-1.00) | 0.049 |  |
| 5-17 years | 1282 | 103.16 (97.59-108.96) | 1.13 (0.93-1.38) | 0.208 |  |
| **Early Post-PCV13 (2011-2014)** | | | | |  |
| 0-1 years | 917 | 500.26 (468.40-533.72) | 1.01 (0.80-1.28) | 0.932 |  |
| 2-4 years | 1114 | 313.14 (295.02-332.09) | 0.77 (0.64-0.93) | 0.007 |  |
| 5-17 years | 1010 | 67.87 (63.74-72.18) | 1.08 (0.88-1.31) | 0.478 |  |
| **Late Post-PCV13 (2015-2019)** | | | | |  |
| 0-1 years | 396 | 424.93 (384.10-468.91) | 0.63 (0.47-0.85) | 0.003 |  |
| 2-4 years | 544 | 285.21 (261.74-310.21) | 0.71 (0.57-0.89) | 0.003 |  |
| 5-17 years | 480 | 56.31 (51.38-61.58) | 0.87 (0.68-1.10) | 0.233 |  |

*Pre-PCV7 (2003-2005). **Where the number of episodes was less than 5 the data are not shown, in accordance with data protection policies. ACP: All-cause Pneumonia; CI: Confidence Interval; IPD: Invasive Pneumococcal Disease; IR: Incidence Rate; IRR: Incidence Rate Ratio; N: Number; NR: Not Reported PCV: Pneumococcal Conjugate Vaccine; PP: Pneumococcal Pneumonia; PY: Person-Years.
